# Does radiolucency really predict loose components in revision shoulder arthroplasty?

**DOI:** 10.1007/s00256-023-04330-7

**Published:** 2023-04-19

**Authors:** Laura E. Streck, Chiara Gaal, Frank Gohlke, Maximilian Rudert, Kilian List

**Affiliations:** 1 Koenig-Ludwig-Haus, Department of Orthopedic Surgery, University of Wuerzburg, Brettreichstrasse 11, 97074 Würzburg, Germany; 2 Department of Shoulder Surgery, Rhoen Clinics, Bad Neustadt, Germany

**Keywords:** Radiolucent lines, Shoulder replacement, Arthroplasty revision, Radiograph

## Abstract

**Objective:**

The number of shoulder arthroplasties is increasing along with the need for revision surgeries. Determining the stability of the implant is crucial in preoperative planning. This study aims to investigate whether radiolucent lines (RLL) in preoperative radiographs predict component loosening.

**Materials and methods:**

Preoperative radiographs of 93 cases in 88 patients who underwent shoulder arthroplasty revision were evaluated regarding the presence of RLL. Correlation analyses were performed for radiographic findings and demographic factors (age, gender, BMI, prior surgeries) compared to intraoperative findings.

**Results:**

The presence of RLL around the humeral component correlated with loosening (*p* < 0.001, Phi 0.511), and the distal zones 3 and 5 showed the strongest correlation (Phi 0.536). While RLL in only one zone did not predict loosening (*p* = 0.337), RLL present in two or more zones showed correlation with loosening (*p* < 0.001). Risk factors associated with loosening were a higher age at the time of revision surgery (*p* = 0.030) and the number of zones with RLL (*p* < 0.001). The glenoid component was loose in 39.0% of the cases; 5.5% of the glenoid components with RLL were stable. Nevertheless, the presence of RLL was highly associated with loosening (*p* < 0.001, Phi 0.603). A longer time between implantation and revision correlated with loosening of the glenoid component (*p* = 0.046).

**Conclusion:**

While RLL do not predict loosening of the implant in general, occurrence in more than one zone correlates with loosening. If located in distal zones and with increasing number of zones with RLL, the correlation becomes even stronger and loosening is more likely.

## Introduction

From 2010 to 2019, the volume of primary shoulder arthroplasties in Germany increased by approximately 14% each year, and a sevenfold increase in case numbers is expected by 2040 [1]. A massive rise in primary implantations is predicted in the United States as well [2]. This will consequently lead to an increase in shoulder arthroplasty revisions.

The reasons necessitating revision surgery are various, including component loosening, periprosthetic fractures, instability, component wear, or periprosthetic infections [3]. The most common cause, according to registry data from the German Shoulder and Elbow Society, is loosening of the humeral or glenoid component [4]. Determining whether an implant is well integrated or loose not only affects the diagnosis but is also crucial for a well-planned strategy for revision surgery for any complications.

If there is consecutive imaging that shows migration over time, loosening seems obvious. Much more common, however, is the sole evidence of radiolucent lines (RLL) between implant and bone. RLL are described in more than 75% of shoulder arthroplasties after 40 months [5] and over 80% after 10 years [6]. The extent to which these RLL are associated with the need for revision surgery is a subject of ongoing debate. The definitions of loosening based on radiographic findings vary in the literature. Kahn et al. defined a “humerus at risk” if RLL are present in three or more zones [7]. Gonzales et al. defined loosening of the shaft as either migration or RLL of > 2 mm around the whole shaft [8]. Loosening was hereby reported in 6% of the cases; however, revision surgery was only required in 1%. Gazielly et al. reported definite radiographic loosening of the glenoid in 15.5%, but only 2.5% required revision surgery [9]. In line with this, the asymptomatic presence of RLL is not necessarily an indication for revision surgery [7-10].

To date, the diagnostic value of RLL to predict the actual clinical stability of an implant in case of a planned revision surgery, regardless of the underlying diagnosis, remains unclear.

This study was designed to answer the following research questions: Can RLL in preoperative radiographs predict loosening of a shoulder arthroplasty prior to revision surgery? What are further risk factors predicting a loose component in shoulder arthroplasty revision?

## Materials and methods

### Patient cohort

**Fig. 1:**
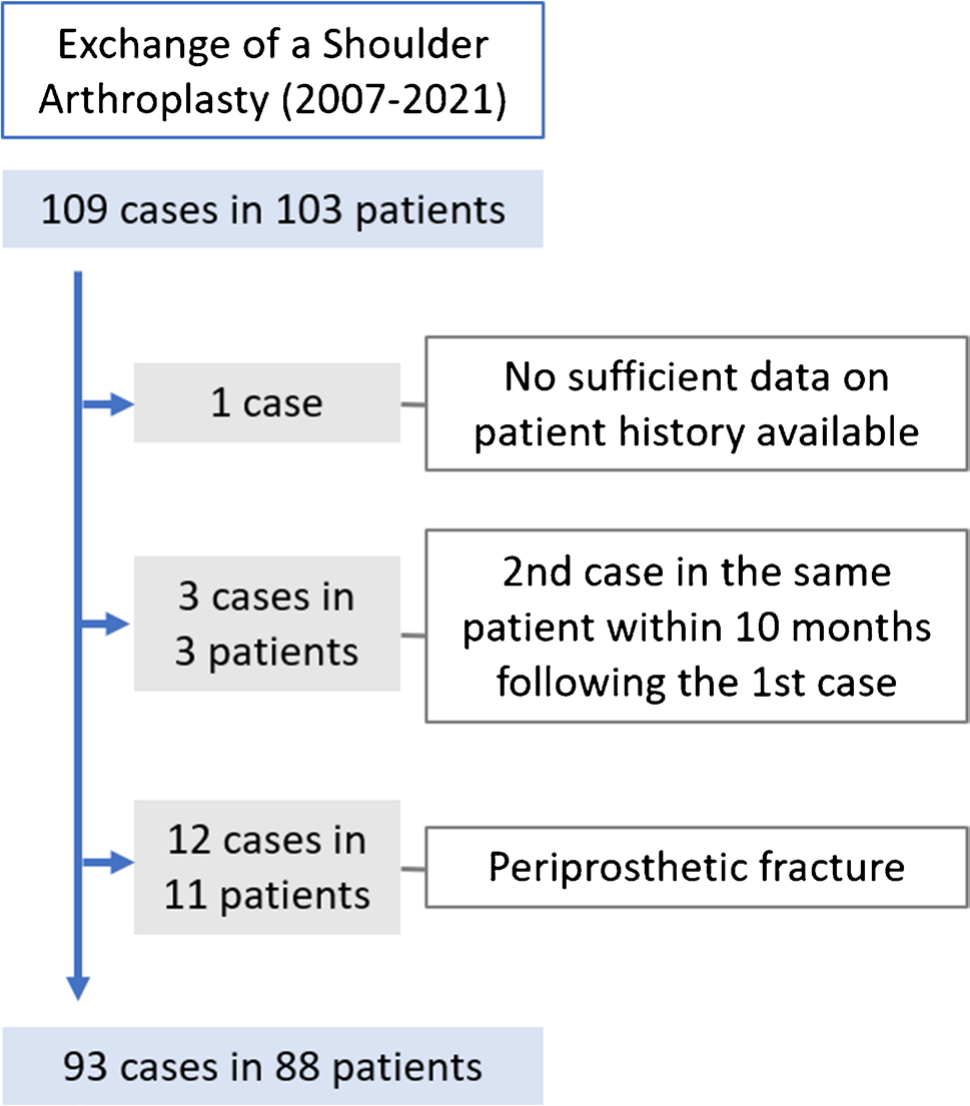
Flowchart presenting the in- and exclusion process of the study cohort. 93 cases were eligible for the study

**Fig. 2:**
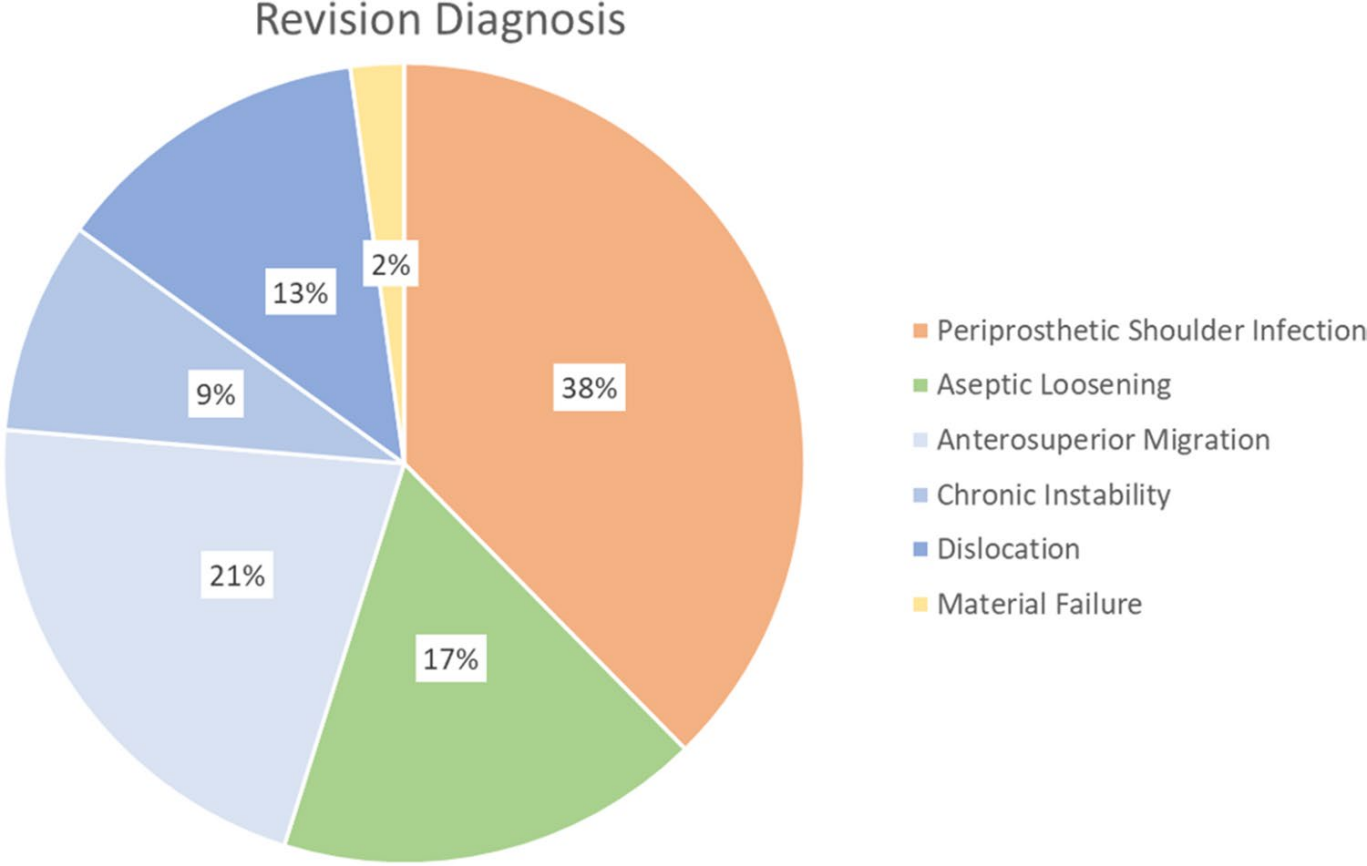
Diagnosis leading to revision surgery in percent of cases. Periprosthetic shoulder infection (38%) was the leading diagnosis, followed by anterosuperior migration of the humeral head (21%) and aseptic loosening (17%)

This is a retrospective study on patients who underwent an exchange shoulder arthroplasty at the authors’ institution between 2007 and 2021. Details on the enrollment with in- and exclusion criteria are provided in the flowchart (Fig. 1). 93 shoulders in 88 patients were eligible for inclusion (74% females, 26% males, 72% right shoulders). Implants were 45 reverse shoulder arthroplasties (RSA), 14 anatomic total shoulder arthroplasties (TSA), 31 hemi-arthroplasties (HSA), and 3 proximal humerus replacements (PHR). Further details on demographics are shown in Table 1 and Fig. 2.

**Table 1:** Demographic information for the study cohort). All cemented glenoids were TSA, all uncemented glenoids were RSA

	number of cases (%)		
fixation type	cemented	uncemented	
glenoid	14 (15.1%)	45 (48.4%)	
humeral component	62 (66.7%)	31 (33.3%)	
	mean	range	standard deviation
age at implantation	67 years	30–87	12
age at revision	71 years	40–91	11
longevity of the implant	55 months	1–350	61

### Radiographic protocol

Radiographs of the shoulder were obtained prior to revision surgery in all cases (mean 1.8 weeks prior to surgery, range 0–12, standard deviation (SD) 3.0). The radiographic assessment included:

1. True antero-posterior view of the shoulder: The patient is standing, with the arm in neutral-zero-position. The scapula is placed flat on the radiographic cassette, and the body is rotated 30–45° in the frontal plane to the direction of the affected shoulder. The beam path is declined 20°. This radiograph provides the orthogonal view of the joint space and shows the greater tubercle on the margin of the radiograph [11].
2. Outlet-view of the shoulder: The patient is sitting, with the arm hanging down. The affected side is rotated 30° away from the stative. The beam path is aligned tangential to the shoulder plate and in the 15–20° cranio-caudal direction [11].

### Definition of radiolucency and loosening

**Fig. 3:**
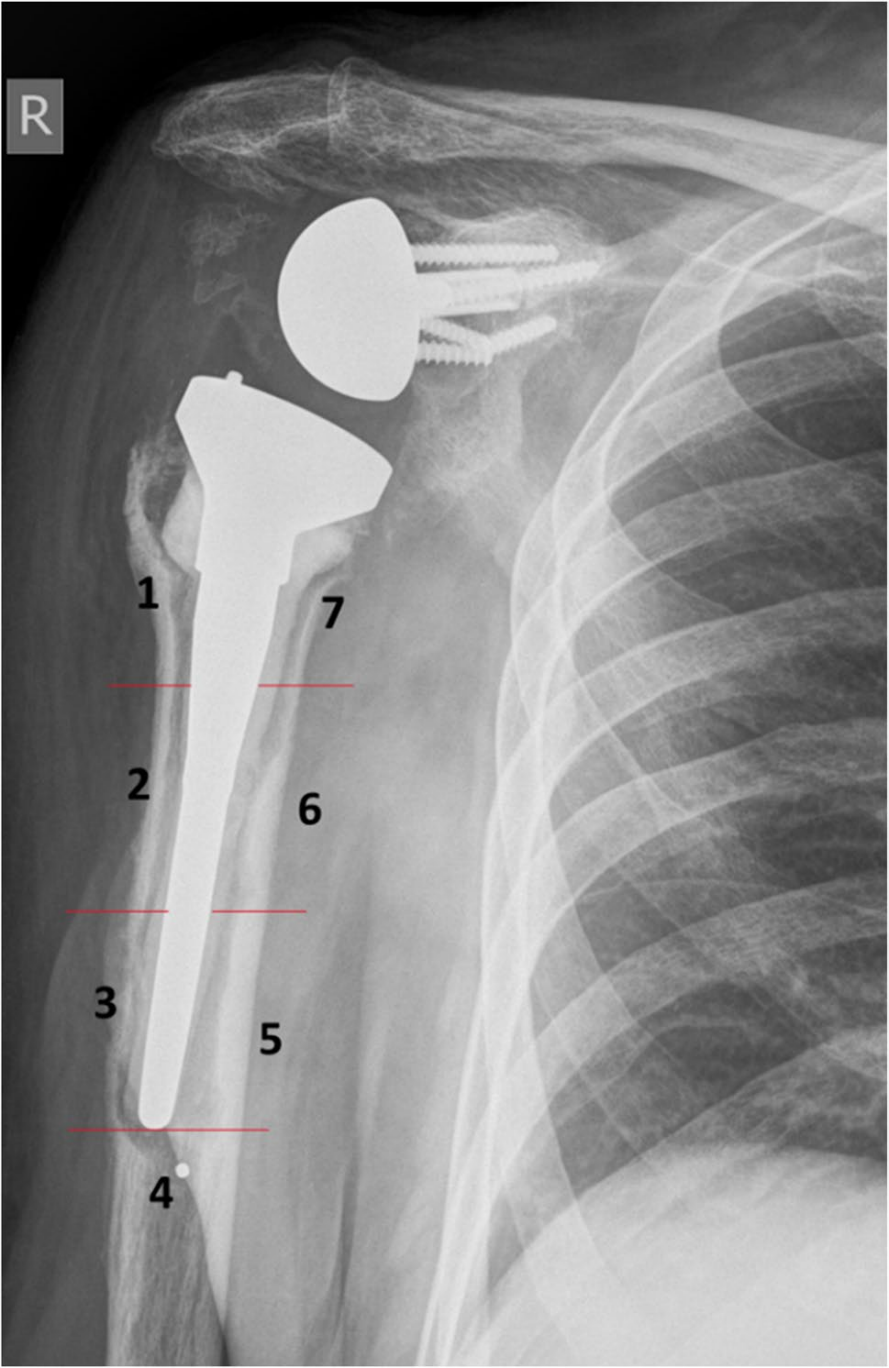
Antero-posterior radiograph of a right shoulder with RSA. The humeral zones according to the classification described by Boileau et al. are labeled 1–7 [5]. In the presented case, RLL are present in all zones

The minimum width of RLL was defined as 2 mm, the localization around the humeral component was classified according to the zones described by Boileau et al. [5]. An example is presented in Fig. 3. The glenoid component was divided into four zones (superior baseplate, inferior baseplate, central peg, screws) in RSA and into three zones (superior, medial, inferior third) in TSA. Loosening of a component was stated according to the intraoperative records.

### Imaging analysis

All images and measurements were obtained from a picture archiving and communication system with planning software (AGFA, Mortsel, Belgium).

All radiographs were initially reviewed by two of the authors (one senior attending and one resident). The senior author repeated assessment of 21 radiographs to detect intra-rater reliability. Cases where the reviewers disagreed were discussed by four of the authors (LS, CG, MR, KL). 

### Statistical analysis

Descriptive statistics were used for demographics and frequencies. A contingence analysis with Pearson chi-square test was performed to test for correlation between categorical variables (dependency loosening on prior implant exchange, type of prosthesis, and type of fixation; presence of RLL). The strength of the correlation was determined with correlation coefficient Phi. Fisher’s exact test was performed to test whether the presence of RLL in one zone correlates with loosening. Binary logistic regression with forward inclusion (Wald) was performed to test for the influence of ordinal/metric variables (age at implantation, age at revision, number of prior surgeries, longevity) on loosening of a component.

Kohen’s Kappa κ was used to measure intra- and interrater reliability on a sample of 21 randomly selected cases. Both intra- and interrater reliability were good, with κ = 0.829, respectively.

Significance level was set at α = 0.050. Statistical analysis was performed with SPSS Statistics 22 (IBM, Armonk, New York, USA). Diagrams were built with Excel version 2202 (Microsoft, Redmont, Washington, USA).

The study has been reviewed by the authors’ Institutional Ethics Committee, and the necessity of approval has been waived due to the study’s retrospective character.

## Results

### Humeral component

The humeral component was found to be stable intraoperatively in 61 cases (65.6%) and was loose in 32 cases (34.4%). There was no difference between an anatomic design (TSA and HSA) and RSA (*p* = 0.378). Uncemented humeral components were loose in 35.5% versus 33.9% of cemented components; this difference was not significant (*p* = 0.877). A history of prior implant exchange was not a risk factor for loosening (*p* = 0.906). Risk factors associated with loosening of the humeral component were a higher age at the time of revision surgery (*p* = 0.030) and the number of zones with RLL (*p* < 0.001). A higher age at the time of implantation showed a tendency to a higher risk for loosening, though this was not significant (*p* = 0.050). The longevity of the implant (time between implantation and revision) did not correlate with loosening (*p* = 0.864). The indication for revision had an impact on the rate of loosening of the humeral component (*p* = 0.018). It was highest for septic loosening (71.4%), followed by aseptic loosening (50.0%), dislocation (33.3%), rotator-cuff related diagnoses (30%), periprosthetic shoulder infections (14.3%), and chronic instability (12.5%).

**Fig. 4:**
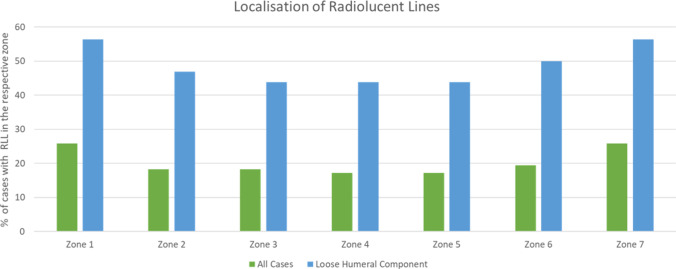
Percentage of cases with a loose humeral component (blue)/all cases (green) with radiographic evidence of RLL for each zone according to the classification described by Boileau et al. [5]

RLL around the humeral component were present in 28 cases (30.1%). The most common localization was the proximal part of the component where RLL were present in zones 1 and 7 in 25.8%, respectively. Figure 4 depicts the distribution of RLL according to the zones described by Boileau et al. [5] in all cases and only cases with intraoperative loosening. The presence of RLL, in general, correlated with loosening (*p* < 0.001, Phi 0.511); however, RLL in only one zone did not predict loosening (*p* = 0.337), but as soon as RLL were present in two or more zones, the correlation with loosening was highly significant (*p* < 0.001).

Moreover, the strength of this correlation varied depending on the localization of the RLL. The distal zones 3 and 5 showed a slightly stronger correlation (Phi 0.536) than the proximal zones 1 and 7 (Phi 0.504).

### Glenoid component

**Fig. 5:**
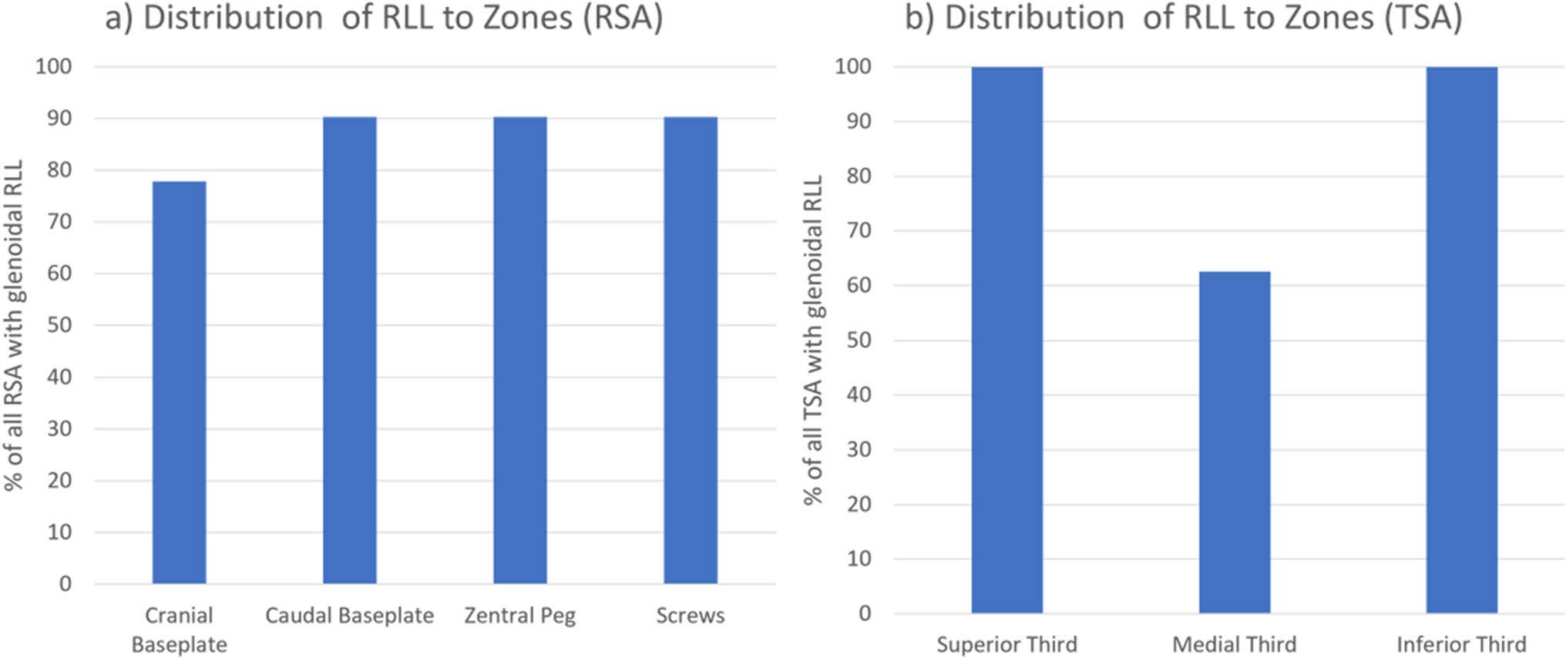
Percentage of cases with precence of RLL in respect of different zones for (**a**) reverse shoulder arthroplasties (RSA) (**b**) anatomic total shoulder arthroplasties (TSA)

The glenoid component was intraoperatively loose in 23 (39.0%) of the cases. 71.4% of the anatomic glenoids but only 28.9% of the RSA baseplates were loose; this difference was significant (*p* = 0.004). A longer time between implantation and revision (longevity) correlated with loosening (*p* = 0.046). Age at implantation, age at revision, history of a prior implant exchange, or the number of prior surgeries did not correlate with loosening (*p* = 0.111/0.110/0.179/0.492). RLL were present in 17 cases (28.8%). Figure 5 depicts the distribution of RLL to the zones. 94.5% of the glenoids with RLL were intraoperatively loose and 5.5% were stable. Therefore, the presence of glenoidal RLL is not an absolute predictor for glenoid loosening; however, it did correlate with high significance (*p* < 0.001, Phi 0.603).

## Discussion

RLL are a common radiographic finding after shoulder arthroplasty. Rates up to 80% after 10 years are described [6]. This study, specifically reporting on cases prior to arthroplasty revision, detected RLL in 29% of the glenoid components and 30% of the humeral components. The relatively low rate may likely be explained by a shorter period between implantation and radiographic evaluation in the current study design. Furthermore, this study defiened RLL at a minimum width of 2 mm [8, 12]; this was not consistant in early studies on this topic [13, 14].

Most publications on RLL focused on a consecutive cohort of patients following shoulder arthroplasties. The current study stands out by directly comparing radiographic to intraoperative findings.

To date, it remains unclear how radiographic findings are associated with actual loosening of the implant. Furthermore, the radiographic definition of loosening is inconsistent in the literature. For example, Melis et al. defined loosening as RLL > 2 mm in more than three zones [12], Gonzales et al. as RLL > 2 mm around the whole shaft, and Kahn et al. saw the “humerus at risk” if there were any RLL in more than three zones [7]. However, only a small percentage of the patients with RLL seems to present symptoms or require revision surgery. In the cohort reported by Gonzales et al., only 2% of the patients underwent revision while 6% where classified as loose according to radiographic evaluation [8]. Therefore, the asymptomatic presence of RLL cannot generally be equated with loosening or an indication for revision surgery [7-10].

The current study focused on revision cases. Even though there was a significant correlation between RLL and loosening, the study showed that 13.1% of the humeral components, positive for RLL were still well fixed, which is a clinically relevant amount. Thus, the appearance of RLL correlates with loosening but does not guarantee for loosening of an implant. The predictive value of RLL was dependent on its localization and magnitude. RLL in only one zone were not associated with loosening (*p* = 0.337); in contrast, RLL in two or more zones were (*p* < 0.001). Furthermore, an increasing number of zones with RLL was associated with a higher risk for a loose implant (*p* < 0.001). While RLL in the proximal zones (1 and 7) were more common in general, RLL in the distal zones (3 and 5) showed an even stronger correlation with loosening (Phi 0.536). Thus, loosening of the humeral component is most likely if the RLL are located distally and involve multiple zones.

As with the humeral component, there is no consensus on the classification of radiographic findings and the definition of loosening in glenoid components. Due to the different fixation techniques in anatomic glenoids versus reverse baseplates, it stands to reason to use different systems for both implant types. However, the huge variety of classifications in the current literature reduces the comparability of the results even more [2, 12, 15]. Over all, loosening rates of up to 65% after 15 years are described [16]. In the current study, RLL were present in 29% of the cases. In TSA, RLL were most likely to occur in the superior and inferior, rather than the central areas. This is in line with the typical loosening mechanism described by Franklin et al. in 1988 as the rocking horse phenomenon [17]. A more even distribution of RLL was found in RSA baseplates, with only the cranial baseplate being affected slightly less frequently. Regardless of the type of implant, RLL were strongly associated with loosening. However, 5.5% of the cases with RLL turned out well fixed.

It is under debate if other imaging techniques might improve the non-invasive detection of implant loosening. Gregory et al. reported RLL in 54 of 55 observations from computer tomography (CT) scans (11 shoulders, 5 observers) and a higher intra-observer reliability compared to radiographs [18]. Yian et al. reported on 47 TSA, 47% of the RLL visualized by CT scans were not seen on plain radiographs [19]. However, none of the studies compared the imaging findings to intraoperative proof of loosening. Mallo et al. compared CT scans of painful shoulder arthroplasties to arthroscopic findings and reported a sensitivity of 70% and a specificity of 75% for the detection of loosening by CT evaluation [20]. Newer techniques such as SPECT/CT are only reported in case series, which are too small to provide reasonable evidence [21]. A recent study by Broden et al. investigated CT micromotion analysis; at a 24-month follow-up, RLL were detected in all glenoid components [22]. However, the functional outcome, assessed by Constant- and Oxford-Shoulder Score, improved over time [22]. Thus, those imaging techniques might provide a more reliable and sensitive option to detect radiographic signs of potential loosening in shoulder arthroplasties, but the clinical relevance of this finding remains questionable.

The current study also aimed to assess patient-related factors predictive of a loose component at revision surgery. Bacle et al. described a significant association between RLL and failed previous arthroplasty [23]. In contrast, our data showed no correlation between a previous exchange of implant and loosening (*p* = 906). The only factors identified to correlate with loosening were a higher age at the time of revision surgery for loosening of the humeral component (*p* = 0.030) and longevity of the implant for loosening of the glenoid component (*p* = 0.046).

The results presented in this study need to be interpreted against the background that only patients that underwent revision surgery were included. Another limitation is the variety in revision diagnoses and the inclusion of different implant designs.

## Conclusion

The findings of this study confirm that RLL are correlated with implant loosening. However, a clinically relevant amount of implants were intraoperatively stable despite the presence of RLL; therefore, RLL do not guarantee loosening. The current results revealed the importance of a detailed analysis of RLL. Implants were more likely to be loose with the presence of RLL in an increasing number of zones and with humeral RLL being located more distally. Having this in mind, conventional radiographs still play a major role in the diagnostic workup and preoperative preparation in revision shoulder arthroplasty. It may be beneficial to also apply this study design of comparing radiographic results to intraoperative findings to alternate imaging techniques and/or combinations of those in order to investigate their clinical relevance.

## Data Availability

The datasets generated during and/or analysed during the current study are available from the corresponding author on reasonable request.

## Abbreviations

CT: Computed tomography
HAS: Hemi shoulder arthroplasty
PHR: Proximal humerus replacement
RLL: Radiolucent line/s
RSA: Reverse shoulder arthroplasty
SD: Standard deviation
SPECT: Single photon emission computed tomography
TSA: Anatomic total shoulder arthroplasty
